# Low-Arousal Speech Noise Improves Performance in N-Back Task: An ERP Study

**DOI:** 10.1371/journal.pone.0076261

**Published:** 2013-10-18

**Authors:** Longzhu Han, Yunzhe Liu, Dandan Zhang, Yi Jin, Yuejia Luo

**Affiliations:** 1 School of Biological Science and Medical Engineering, Beihang University, Beijing, China; 2 National Key Laboratory of Cognitive Neuroscience and Learning, Beijing Normal University, Beijing, China; 3 Institute of Affective and Social Neuroscience, Shenzhen University, Shenzhen, China; Harvard Medical School/Massachusetts General Hospital, United States of America

## Abstract

The relationship between noise and human performance is a crucial topic in ergonomic research. However, the brain dynamics of the emotional arousal effects of background noises are still unclear. The current study employed meaningless speech noises in the n-back working memory task to explore the changes of event-related potentials (ERPs) elicited by the noises with low arousal level vs. high arousal level. We found that the memory performance in low arousal condition were improved compared with the silent and the high arousal conditions; participants responded more quickly and had larger P2 and P3 amplitudes in low arousal condition while the performance and ERP components showed no significant difference between high arousal and silent conditions. These findings suggested that the emotional arousal dimension of background noises had a significant influence on human working memory performance, and that this effect was independent of the acoustic characteristics of noises (e.g., intensity) and the meaning of speech materials. The current findings improve our understanding of background noise effects on human performance and lay the ground for the investigation of patients with attention deficits.

## Introduction

Background noise usually interferes with cognitive processing and has detrimental effects on mental and physical health [1]–[2]. The relationship between noise and human performance is therefore a crucial topic in ergonomic research [3]. A vast amount of effort has been devoted to investigating the noise effect on human performances, such as attention and memory [3]–[5]. For example, using event-related potential (ERP) technology, researchers found that the latency of the P3 component was delayed during white noise presentation when compared with silent condition in an oddball paradigm [6]–[7]. In a visual-spatial attention task, Trimmel and Poelzl [8] found the reaction time (RT) was prolonged and the DC-potential shifted towards positivity in a noise condition compared to a silent condition. One of the cognitive mechanisms that possibly underpin noise effects on human performance is working memory degradation [9]. It has been observed that participants with low working memory capacity were more susceptible to auditory distracters [4]. According to Baddeley & Hitch's working memory model [10], when participants perform certain memory-related tasks, working memory rehearses information in an auditory format. As a result, background noise would disrupt material held in the phonological loop of working memory [11].

Previous studies have confirmed that the noise effect on human performances may vary according to the type of noise format [3]. Speech is one of the most frequently employed noise format in ergonomic experiments. Humans are especially attuned to speech [12]; even irrelevant speech is monitored to some degree, as evidenced by the cocktail party effect [13]. It has been observed that participants performed worse in the serial recall task when the noise was irrelevant speech; in contrast, white noise did not significantly affect the performance [14]. The phenomenon that speech noise is more disruptive than non-speech noise may be due to either the physical properties or the meaning of speech [14]–[15]. Jones and Morris [15] showed that the speech intensity and the exposure duration were the most influential moderators of the interruptive effect of speech noise; however, Salamé and Baddeley [14] indicated that the noise effect may come primarily from the instinct meaning of speech.

Noise is able to change the arousal of subjects, as reflected by muscle tension, skin resistance, blood pressure, pulse rate, and metabolic rate [16]. Several studies have suggested that the noise disruption effects on task performance may depend on the level of arousal induced by noises [17]–[18]. For example, compared to silent environment, participants performed better in the Rod-and-Frame test and in the Stroop test when listening to low arousal noises [19]–[20]. A popular theory explaining the noise effects on arousal is the attentional narrowing mechanism [21]. It suggests that noises increase arousal and the increased arousal, in turn, decreases the breadth of attention [19], [22]. More specifically, when at a relatively low arousal level, proper attentional narrowing helps to exclude irrelevant cues and thus would facilitate performances. While the noise-induced arousal reaches beyond an appropriate level, further attentional narrowing may impair the processing of task-relevant cues and the individual performance would decline accordingly [23]. However, most previous studies on noise arousal effects have examined the intensity dimension of the noise. In our opinion, although noise with higher intensity may induce higher arousal level of participants, the listeners' arousal can be manipulated by not only stimulus intensity but also the emotional arousal itself (i.e., independent of stimulus intensity) [5].

The present study aimed to investigate the ERP correlates of the effects of arousal-dependent speech noise on working memory. In order to focus on the emotional arousal instead of the acoustic features (e.g., intensity) of speech noises, and to exclude the potential influence of speech meaning on performance, this study employed meaningless pseudo-sentences with matched acoustic features as background noises in the classic n-back working memory task (n = 1 or 3). We hypothesized that the emotional arousal level of speech noises could affect both the behavior and ERP signals of participants; compared to silent and high-arousal conditions, the low-arousal speech noises would improve the memory performance.

## Materials and Methods

### Participant

Twenty-two healthy subjects (12 females; age range  = 18 to 24, mean  = 21.3 years) were recruited from Beijing Normal University as paid participants. All subjects were right-handed and had no history of neurological diseases. All were free of regular use of medication or other nonmedical substances which potentially affect the central nervous system. They had normal hearing and normal or corrected-to-normal vision. They gave their written informed consent prior to the experiment. The experimental protocol was approved by the local ethics committee (Beijing Normal University) and was in compliance with the ethical guidelines of the American Psychological Association.

### Materials

Task-irrelevant speech materials were consisted of 60 pseudo-sentences (30 fearful and 30 happy sentences), which were selected from the validated database of Chinese vocal emotional stimuli [24]. These pseudo-sentences were composed of pseudo content words conjoined by real function words, rendering them semantically meaningless but ensuring that the phonetic/segmental and suprasegmental properties were appropriate to native Mandarin speakers/listeners (e.g., in English: The fector jabbored the tozz). The 30 fearful (valence  = 1.31±0.21; mean ± SD) and 30 happy pseudo-sentences (valence  = 4.02±0.14) were divided into low vs. high arousal groups according to their arousal ratings on a 5-point scale (where 1 referred to “very low arousal” and 5 referred to “very high arousal”). In particular, the 15 fearful and the 15 happy pseudo-sentences with the highest arousal ratings out of the 30 fearful and the 30 happy pseudo-sentences, respectively, were put into the high arousal group. The rest of pseudo-sentences (15 fearful and 15 happy ones) were put into the low arousal group. The two arousal groups of emotional pseudo-sentences were carefully matched for valence, recognition rate, duration, and fundamental frequency (refer to Table 1). All the acoustic and emotional ratings were provided by the developers of the database of Chinese vocal emotional stimuli [24].

**Table 1:** Characteristics of low- and high-arousal speech materials.

Characteristic^a^	low arousal		high arousal		t-test	
	mean	SD	mean	SD	*t*(58)	*p*
arousal (5-point scale)	2.40	0.26	3.97	0.25	−23.7	<.001
valence (5-point scale)	2.69	1.38	2.65	1.39	0.12	.906
recognition rate (*Hu* score)	0.57	0.15	0.60	0.17	−0.54	.592
duration (s)	1.58	0.17	1.53	0.20	0.85	.397
f0 (normalized value)^b^	1.14	0.08	1.18	0.11	−0.83	.407

a Data were from [24].^ b^ Fundamental frequency.

### Procedure

Participants were seated in a dimly lit and sound-attenuated room. Visual stimuli were presented on a LCD monitor (refresh rate  = 60 Hz) at a viewing distance of 100 cm. Stimulus display and behavioral data acquisition were conducted using E-Prime software (Version 1.1, Psychology Software Tools, Inc., Pittsburgh, PA). The mean background noise level (without speech material presentation) was 30 dB sound pressure level (SPL) (Brüel & Kjær, Nærum, Denmark; sound level meter type 2209; octave filter type 1603).

Participants were told to memorize cardinal numbers presented on the screen and ignore the irrelevant speech noises during the experiment. The arousal effects of speech noises were investigated in three sessions in the experiment. Besides low- and high-arousal sessions, which were studied by persistently presenting low- and high-arousal pseudo-sentences until the current session was ended, a silent session was also designed with no speech noise presented during the n-back task (no-arousal condition in this study). In low- and high-arousal sessions, the 30 pseudo-sentences might be sampled more than once by the E-Prime program; sampled pseudo-sentences were concatenated in a random order and were presented with equal intensity (60 dB SPL). The order of the three sessions was pseudo-randomized across subjects. Each session was comprised of two blocks with fixed levels of working memory load (1- and 3-back tasks). The 1-back task was always performed first, followed by the block of 3-back task. Blocks were separated by self-terminated breaks.

**Figure 1:**
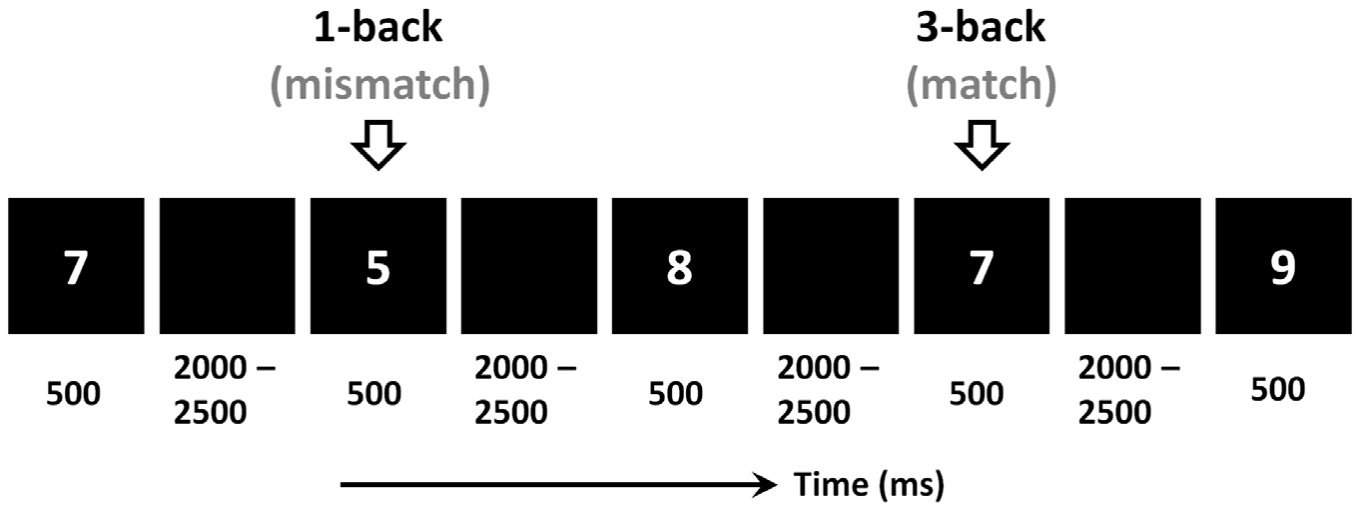
Illustration of the n-back paradigm (n = 1 and 3) in this study.

As illustrated in Figure 1, each trial began with the presentation of a randomly chosen cardinal number (from 0 to 9) for 500 ms (1.7°×1.1° size in the centre of a screen), followed by a 2000-to-2500-ms blank screen. Participants had to match the current stimulus with the previous stimulus (1-back task) or with the stimulus three presentations earlier in the sequence (3-back task). Subjects were instructed to respond as quickly and accurately as possible, with a “yes” key for a match and a “no” key for a mismatch. The ratio of match to mismatch was 1∶1. Participants were instructed to press the “F” and “J” on the computer keyboard with their right and left index fingers. The assignment of keys to “yes” and “no” responses was counterbalanced across participants. There were 60 trials in each block. Prior to the formal experiment, participants performed at least 25 practice trials for each memory load to familiarize themselves with the n-back task. The practice phase would be prolonged if necessary, until 80% correct responses were achieved in both the 1- and 3-back tasks.

### EEG recording and preprocessing

Brain electrical activity was recorded referentially against left mastoid and off-line re-referenced to average reference, by a 64-channel amplifier with a sampling frequency of 250 Hz (NeuroScan Inc., Herndon, USA). Besides electrooculogram electrodes, a 60-channel electroencephalography (EEG) data were collected with electrode impedances kept below 5 kΩ. Ocular artifacts were removed from EEGs using a regression procedure implemented in Neuroscan software (Scan 4.3).

The data analyses and result display in this study were performed using Matlab R2011a (MathWorks, Natick, USA). The recorded EEG data were filtered with a 0.05–30 Hz finite impulse response filter with zero phase distortion. Filtered data were segmented beginning 200 ms prior to the onset of cardinal number figures and lasting for 1000 ms. All epochs were baseline-corrected with respect to the mean voltage over the 200 ms preceding the onset of the figures, followed by averaging in association with experimental conditions.

### ERP analysis

In the present study, we focused on the ERPs elicited by low (1-back) and high (3-back) memory load and in low- and high-arousal speech conditions. This study analyzed the potentials of occipito-temporal P1 and N1, fronto-central P2, and parietal P3 components across different sets of electrodes according to grand-mean ERP topographies [25]–[27]. Time windows for mean amplitude calculation were centered at the peak latencies of ERP components in grand-mean waveforms, with a shorter window length for early components and a longer length for late components. The mean amplitudes of P1 and N1 component were calculated at PO5, PO6, PO7, and PO8 (time window: 105–115 ms for P1; 160–180 ms for N1). The mean amplitude of P2 component was analyzed at the FC1, FCz and FC2 electrode sites (time window: 160–180 ms). The mean amplitude of P3 component was analyzed at the P1, Pz and P2 electrode sites (time window: 350–400 ms).

### Statistics

Statistical analyses were performed using SPSS Statistics 20.0 (IBM, Somers, USA). Descriptive data were presented as mean ± standard deviation (SD). The significance level was set at 0.05. A two-way repeated-measures ANOVA was performed on the reaction time (RT) and accuracy rate (ACC) measurements with memory load (1-back vs. 3-back) and speech arousal level (silent, low arousal, and high arousal) as the two within-subjects factors. A three-way repeated measures ANOVA on the mean amplitudes of P1, N1, P2, and P3 components were conducted with memory load, arousal level, and electrodes (refer to the ERP analysis subsection for specified electrode sites of different ERP components) as within-subjects factors. Greenhouse-Geisser correction for ANOVA tests was used whenever appropriate. Post-hoc testing of significant main effects was conducted using Bonferroni method. Significant interactions were analyzed using simple effects models. Partial eta-squared (partial η^2^) was reported to demonstrate the effect size in ANOVA tests, where 0.05 represents a small effect, 0.10 indicates a medium effect, and 0.20 represents a large effect. For the sake of brevity, effects that did not reach significance have been omitted.

## Results

### Behavior

The behavioral data of RT and ACC are listed in Table 2. A repeated-measures 2×3 ANOVA was performed with memory load and arousal level as the two within-subjects factors, and with RT as the dependent variable. The main effect of memory load was significant (*F*(1, 21)  = 28.6, *p* = .000, partial η^2^  = 0.577). Subjects responded faster in the 1-back task (305.3±125.9 ms) than in the 3-back task (516.7±143.1 ms). The main effect of arousal level was significant (*F*(2, 42)  = 3.9, *p* = .031, partial η^2^  = 0.156). Subjects responded faster in the low arousal condition (368.3±129.2 ms) than in the silent (429.8±130.8 ms, *p* = .044) and high arousal conditions (434.8±139.0 ms, *p* = .089), while the latter two conditions showed no RT difference (*p* = 1.000).

**Table 2:** Behavioral results of the 22 subjects (data are presented as mean±SD).

measure	silent (no arousal)		low arousal		high arousal	
	1-back	3-back	1-back	3-back	1-back	3-back
RT (ms)	336±126	524±221	260±110	477±207	321±173	549±232
ACC (%)	88±10	74±14	86±11	75±11	86±13	74±10

A repeated-measures 2×3 ANOVA was performed with memory load and arousal level as the two within-subjects factors, and with ACC as the dependent variable. The main effect of memory load was significant (*F*(1, 21)  = 36.9, *p* = .000, partial η^2^  = 0.637); the ACC was larger in the 1-back condition (86.6±12.1%) than in the 3-back condition (74.3±12.2%).

### ERP

**Figure 2:**
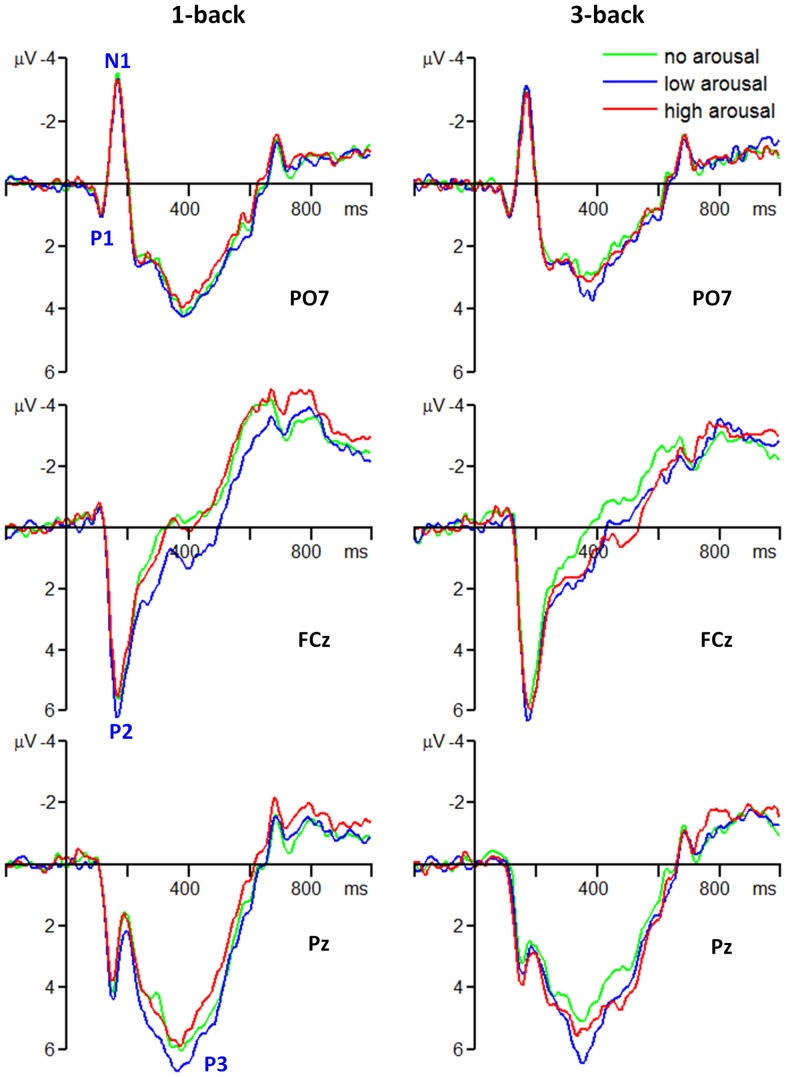
Grand average ERPs of the occipito-temporal P1 and N1, fronto-central P2, and parietal P3 components at typical electrode sites.

The results of the ANOVAs showed that there were no significant main or interaction effect for P1 amplitudes (0.75±1.68 μV; Figure 2, top plots).

The main effect of memory load was significant for N1 amplitudes (*F*(1, 21)  = 4.88, *p* = .038, partial η^2^  = 0.189). The N1 was larger in 1-back task (−2.93±3.23 μV) than in 3-back task (−2.60±3.27 μV) (Figure 2, top plots).

**Figure 3:**
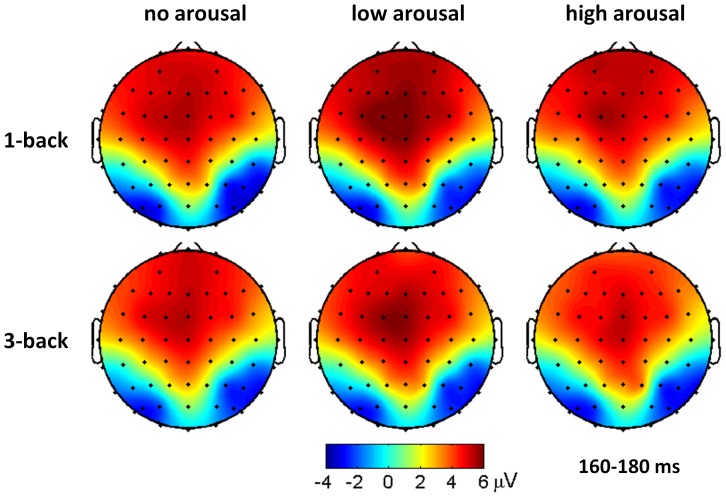
Grand average ERP topographies of the occipito-temporal N1 and fronto-central P2 components across different conditions.

The main effect of arousal level was significant for P2 amplitudes (*F*(2, 42)  = 3.29, *p* = .047, partial η^2^  = 0.135). The P2 was larger in the low arousal condition (5.73±4.37 μV) than in the no arousal (5.18±4.19 μV, *p* = .153) and the high arousal conditions (5.10±3.90 μV, *p* = .084) while the latter two conditions showed no P2 amplitude difference (*p* = 1.000) (Figure 2, middle plots). The main effect of electrode site was significant for P2 amplitudes (*F*(2, 42)  = 8.46, *p* = .001, partial η^2^  = 0.287). The P2 was smaller at FC2 electrode (4.94±3.96 μV) than at FC1 (5.50±4.27 μV, *p* = .028) and FCz electrodes (5.56±4.24 μV, *p* = .001) (Figure 3).

**Figure 4:**
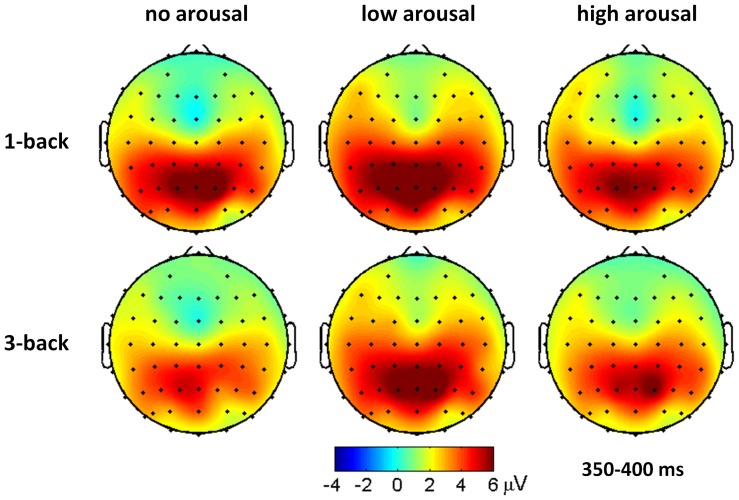
Grand average ERP topographies of the parietal P3 components across different conditions.

The main effect of memory load was significant for P3 amplitudes (*F*(1, 21)  = 5.46, *p* = .029, partial η^2^  = 0.206). The P3 was larger in 1-back task (6.29±4.16 μV) than in 3-back task (5.50±4.07 μV) (Figure 2, bottom plots; Figure 4). The main effect of arousal level was significant for P3 amplitudes (*F*(2, 42)  = 7.50, *p* = .002, partial η^2^  = 0.263). The P3 was larger in the low arousal condition (6.52±3.24 μV) than in the no arousal (5.46±4.07 μV, *p* = .005) and the high arousal conditions (5.70±3.82 μV, *p* = .028) while the latter two conditions showed no P3 amplitude difference (*p* = 1.000) (Figure 2, bottom plots; Figure 4).

## Discussion

This study examined the emotional arousal effect of speech noises in the n-back working memory task. In the experiment, the task-irrelevant speech was meaningless and the physical properties of speech noises were carefully controlled. Considering that the very early P1 component (∼110 ms) was sensitive to the physical characteristics of exogenous stimuli [28], no difference in P1 amplitudes across silent, low-, and high-arousal conditions suggested that the ERP results were comparable between noise vs. silent conditions and between low- and high-arousal conditions.

The current data supported our hypothesis that low-arousal speech noises improved the memory performance. Compared to silent and high arousal conditions, subjects responded faster had larger P2 and P3 amplitudes in the low arousal condition. Our results were in line with the previous study of intensity-dependence effects of background noise [29], which showed that the noise effects at quiet (<40 dB), 70, and 100 dB conditions were U-shaped, with 70 dB background noise improving performance in the maze task. The equivalent effect of the silent and the high-arousal conditions on working memory might be due to different reasons. In silent background condition, participants were not able to exclude the interference of irrelevant information (e.g. equipments and furniture in the laboratory); whereas in high-arousal condition, increased arousal caused increased attentional narrowing so that task-relevant information was also excluded and the performance was thus impaired [23]. More interestingly, the ERP pattern in the current n-back working memory study was very similar with the event-related field (ERF) pattern observed in some auditory perception experiments [30], [31]. For instance, Alain et al. [30] found that larger amplitudes of auditory evoked field (AEF) components were elicited when the target stimuli were embedded in low-level background white noise than in the no-noise condition and in the intermediate-level noise condition. Considering that previous studies [15], [29]–[31] mainly focused on the intensity dimension of noises, the current study provided a strong electrophysiological evidence that the arousal effects of noises can be independent from their acoustic characteristics (e.g., intensity).

The noise arousal findings of this study may help improve the understanding of the characteristics of patients with attention deficits, who are typically with a low dopamine level and thus in a very low arousal state [32]. Studies found that while the exposure to high-intensity white noises worsened the performance of normal school children, it significantly improved the performance in inattentive children [33]. Also, the current study offered some novel implications for the therapy of patients with attention deficits. Since the emotional arousal dimension is independent from the intensity, it is not necessary for therapists to manipulate the intensity of auditory therapeutic materials so as to induce different attention levels of patients; instead, therapists can present speech noises with different emotional arousal ratings, which seem to be more appealing to patients, especially those who are young and hyperactive.

Our result also indicated that the manipulation of working memory load was effective, with slower RT and smaller N1 and P3 components in 3-back condition than in 1-back condition. The P3 amplitude decreased significantly as working memory load increased, which was consistent with previous findings in n-back [34]–[35] and other working-memory-related tasks [36]–[37]. It is assumed that the n-back task consists of two distinct cognition phases: the information maintaining and updating phase (encoding, search and selection of the information) and the matching phase (comparison of the current stimulus with the target stimulus in the memory buffer) [27]. The cognitive resource required in information maintaining and updating phase varies parametrically with memory load, while the resource demand of matching phase is kept constant [27], [38]. The P3 amplitude decreased with increasing memory load, reflecting reallocation of cognitive resource from the matching phase to memory maintenance phase [27], [39]. In addition, we also found that the increase in working memory load was associated with a decrease of the N1 amplitude, which was in agreement with the N1 tendency in previous ERP studies [40]–[41]. In addition, similar results were also observed in fMRI studies [42]–[43]. For instance, Savini et al. [42] found in a somatosensory n-back task that the primary somatosensory activity decreased with increasing n. However, our result was inconsistent with a previous study of SanMiguel et al. [44], which showed that the N1 was not affected by working memory load in a simple visual classification task when subjects ignored contingent irrelevant sounds. The discrepancy between these two studies may be due to different working memory loads investigated in the experiment: we compared the 1-back and the 3-back tasks in this study while the 0-back (i.e. no memory needed) and the 1-back tasks were used in the study of SanMiguel et al. [44].

One limitation of this study was that we failed to record the physiological parameters sensitive to arousal levels such as skin conductance response (SCR). However, as the current experiment required participants to use both hands, the SCR could not be reliably measured. Even though, we believed that the ratings of speech materials (evaluated by another group of participants) insured, to some extent, the validity of arousal inducing in experimental participants.

## Conclusions

The present study is a pilot neuroergonomic research that focused on the neural correlates of the emotional arousal effect of background noise on working memory. Results showed that task-irrelevant speech noises with low arousal ratings were associated with enhanced memory performance and increased ERP voltages in the n-back task. Compared to the silent and the high arousal conditions, participants responded more quickly and having larger P2 and P3 amplitudes in the low arousal condition. Our results suggested that the emotional arousal dimension of task-irrelevant speeches affects human performance, which is independent of the acoustic property of noises such as intensity. While high-arousal noises impair memory performance, low-arousal noises may effectively improve the performance. The current findings improve our understanding of background noise effects on human performance and lay the ground for the investigation of patients with attention deficits.
